# Re-Constructing Historical Adélie Penguin Abundance Estimates by Retrospectively Accounting for Detection Bias

**DOI:** 10.1371/journal.pone.0123540

**Published:** 2015-04-24

**Authors:** Colin Southwell, Louise Emmerson, Kym Newbery, John McKinlay, Knowles Kerry, Eric Woehler, Paul Ensor

**Affiliations:** Australian Antarctic Division, Department of the Environment, 203 Channel Highway, Kingston, Tasmania, 7050, Australia; Phillip Island Nature Parks, AUSTRALIA

## Abstract

Seabirds and other land-breeding marine predators are considered to be useful and practical indicators of the state of marine ecosystems because of their dependence on marine prey and the accessibility of their populations at breeding colonies. Historical counts of breeding populations of these higher-order marine predators are one of few data sources available for inferring past change in marine ecosystems. However, historical abundance estimates derived from these population counts may be subject to unrecognised bias and uncertainty because of variable attendance of birds at breeding colonies and variable timing of past population surveys. We retrospectively accounted for detection bias in historical abundance estimates of the colonial, land-breeding Adélie penguin through an analysis of 222 historical abundance estimates from 81 breeding sites in east Antarctica. The published abundance estimates were de-constructed to retrieve the raw count data and then re-constructed by applying contemporary adjustment factors obtained from remotely operating time-lapse cameras. The re-construction process incorporated spatial and temporal variation in phenology and attendance by using data from cameras deployed at multiple sites over multiple years and propagating this uncertainty through to the final revised abundance estimates. Our re-constructed abundance estimates were consistently higher and more uncertain than published estimates. The re-constructed estimates alter the conclusions reached for some sites in east Antarctica in recent assessments of long-term Adélie penguin population change. Our approach is applicable to abundance data for a wide range of colonial, land-breeding marine species including other penguin species, flying seabirds and marine mammals.

## Introduction

Ecologists are increasingly turning to historical abundance data to understand past changes in animal abundance and more broadly the ecosystems in which animals occur. If assessed correctly, historical abundance data can provide insights into the rates and causes of past population change and make important contributions to ecological and management studies [1]. In practice however, developing reliable ecological or management interpretations from temporal abundance data can be difficult because most population counts are subject to measurement or estimation error. These errors occur when it is not possible to directly and accurately enumerate all individuals in the population [2].

There is now widespread recognition that counts of animal populations are often subject to detection bias, and that the extent of bias may vary over time and space due to aspects of a species’ biology, the type of counting methods used, and the environmental conditions under which a population survey is undertaken [3,4]. This recognition has led to the development of a general framework for abundance estimation that explicitly accounts for detection bias and its uncertainty [3,5,6], new methods for estimating detection bias, and calls for ecologists to estimate and account for bias and uncertainty when estimating animal abundance [7]. While these methodological developments are now being increasingly accepted and used, there is a wealth of historical population count data in the literature that were collected before these developments. These historical abundance data may, in their original published form, have inherent unrecognised and therefore unaccounted biases and uncertainties that could confound reliable interpretation. Developing approaches to improve interpretation of historical data may therefore allow more reliable assessment of extremely valuable long-term abundance data.

There are two forms of detection bias associated with animal abundance counts: (1) perception bias, where animals are missed even though they are within an observers’ view, and (2) availability bias, where animals are missed because they are outside the observers’ view and hence not available for detection [8]. Availability bias is generally more difficult to estimate because it requires collection of additional data using methods external to the counting effort [9]. Land-breeding marine predators, which are considered to be useful and practical indicators of marine ecosystem change due to their dependence on marine prey and their relative accessibility and ease of study when breeding on land [10–12], are particularly prone to availability bias because variation in breeding phenology and breeding success can lead to variable attendance of animals at breeding colonies where population surveys are usually undertaken.

In the Southern Ocean, penguins have been subject to the most temporally and spatially intense population survey effort [13] and have provided the most widely used predator data for inferring marine ecosystem change. Historical penguin abundance data have been interpreted to reflect long-term marine ecosystem change in the Western Antarctic Peninsula region [14–17], an inferred ‘regime shift’ in the marine ecosystem in east Antarctica [18–20], and environmental change and resource extraction impacts in the Ross Sea [21–23]. One feature of historical penguin population count data in Antarctica is that the earliest counts were made in an era of basic biological discovery when there was little quantitative data on breeding biology and phenology of relevance to the issue of availability bias. This, combined with the fact that the challenging logistics of undertaking research in Antarctica often determines when population surveys are conducted, led to historical population counts being made at varying times within the breeding season that were often sub-optimal for abundance estimation. Although researchers attempted to account for phenology-related availability bias in compendia of historical population estimates (e.g. [24]), these efforts were constrained by the scarcity of detailed breeding phenology and attendance data which requires laborious, repeated observations at remote locations, and the adjustment methods lacked statistical rigour. This legacy is inherent to varying degrees in published historical penguin abundance estimates and may affect their interpretation. In comparison, historical penguin population counts are less affected by perception bias because of the species’ relatively large body size and tendency to breed above-ground in open habitats.

Recently, statistically rigorous approaches have been developed to account for phenology-related availability bias in historical penguin count data [25–28]. The approach in [25] uses hierarchical Bayesian models of timing in clutch initiation and nest attrition rates to correct counts of nests made at sub-optimal times. The models were developed from available data on clutch initiation and nest attrition in the Antarctic Peninsula, and have been applied to historical and contemporary counts of penguin nests and chicks across the Antarctic Peninsula to assess spatio-temporal population change in that region [16]. The approach in [26–28] uses data obtained from remotely operating cameras on attendance of a broader range of population objects at breeding sites (adults, nests and chicks) and non-parametric bootstrap methods to develop and apply correction factors to population counts of adults, nest or chicks. Here we apply the latter approach to historical Adélie penguin (*Pygoscelis adeliae*) count data from east Antarctica where (1) few data on phenology or attendance were available prior to our study, (2) many population counts are of adults as well as nests and chicks, and (3) there has been no re-assessment of historical population data. Our re-constructed abundance estimates, which take account of biases and uncertainties, alter the some of the conclusions about long term population change in a recent global assessment of this species that compared historical published estimates with recent satellite-derived estimates [29] and in an earlier assessment of population trends in Antarctic seabirds [30]. The methods and findings have relevance beyond the selected focal species and if applied more broadly could lead to improved interpretation and understanding of past processes operating at population and ecosystem levels in the marine environment.

## Materials and Methods

### Historical abundance data

We re-constructed historical abundance estimates for population count data collected under the Australian Antarctic Program over four decades from the 1950s to 1980s and published in widely used compendia [13,31]. The re-construction analysis focussed on estimates that did not specifically include collection and application of availability adjustment data as an integral part of the survey design and estimation process. We focussed on these estimates in the analysis because we considered they were potentially subject to availability bias. This criterion excluded only two abundance surveys [32,33] from the analysis. Abundance estimates from these two studies should be unbiased because the issue of availability bias was directly addressed. Details of the re-construction steps used in the analysis are below, but we first outline aspects of Adélie penguin population dynamics at their breeding sites that are relevant to the issue of availability bias.

### Adélie penguin breeding biology and attendance at the breeding site

**Fig 1:**
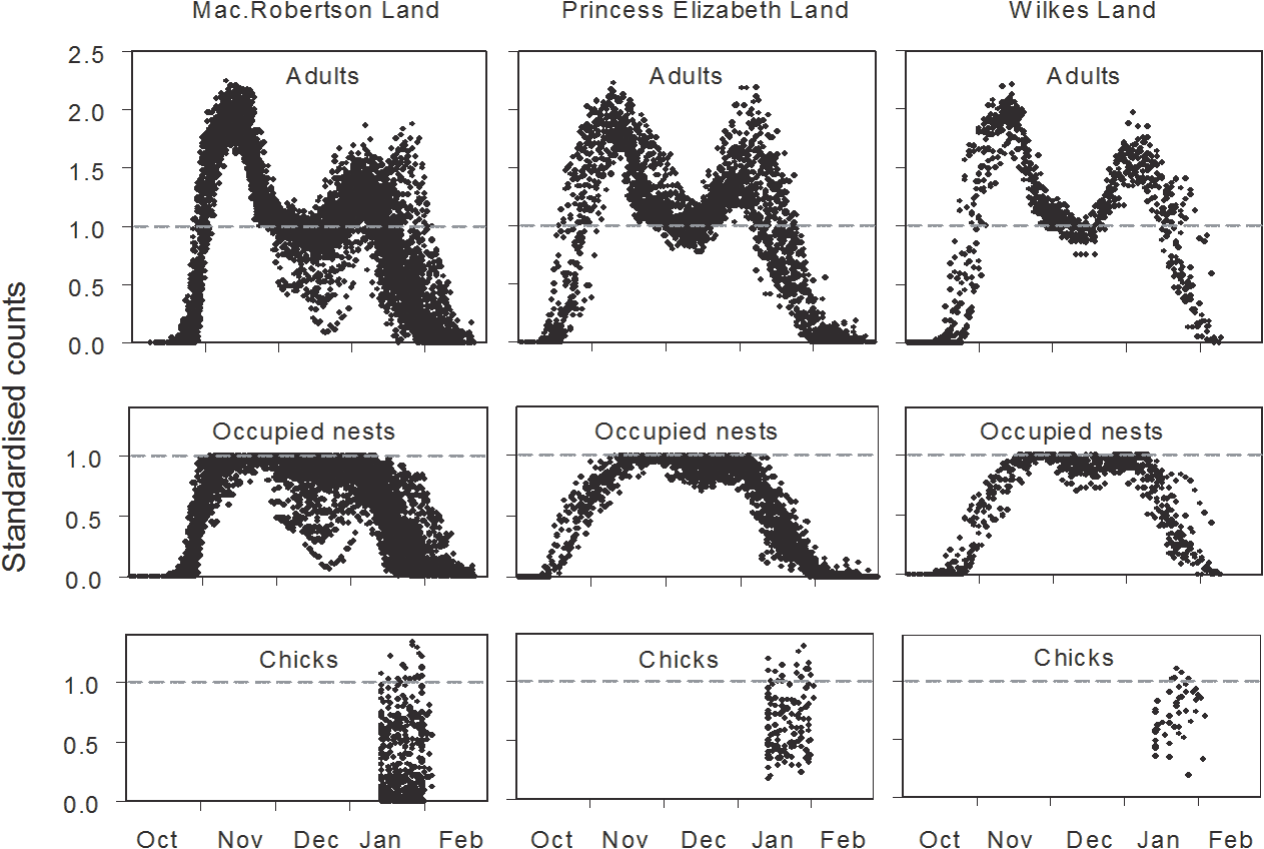
Standardised time series counts of Adélie penguin adults, occupied nests and chicks at breeding sites. Counts were obtained from images taken by time-lapse cameras at 20 locations in three regions of east Antarctica and are standardised to the maximum number of occupied nests. The envelope of points in each plot indicates spatio-temporal variation from time series over multiple camera locations and years (Mac.Robertson Land: 60 time series from 9 cameras from 2005/06 to 2013/14; Princess Elizabeth Land: 28 time series from 6 cameras from 2008/09 to 2013/14; Wilkes Land: 13 time series from 5 cameras from 2011/12 to 2013/14).

Adélie penguins in east Antarctica start arriving at their land-based breeding colonies around mid-October and numbers peak in early November (Fig 1). Many of these penguins form breeding pairs, establish nests and produce chicks, but a proportion either don’t attempt to breed or fail early in their breeding attempt. Penguin numbers in the colonies subsequently decrease from mid-November as females leave to forage shortly after egg lay. Non-breeders and failed breeders also leave the colony at this time, and by late November to early December the colony population is comprised almost entirely of a single male incubating eggs at each nest [34,35]. The number of occupied nests and attending adults generally remains constant through to late December, although both can decrease if nests fail and attending adults leave the colony. From late December to mid-January there is an influx of non-breeders and failed breeders, and some nests that had previously been abandoned are re-occupied. Chicks reach independence around mid-January, and from this time onwards the number of adults and occupied nests decreases as adults spend more time foraging to provision their chicks.

### Availability bias and abundance estimation

A widely accepted metric for Adélie penguin breeding abundance surveys is the number of occupied nests present during incubation when the population is comprised almost entirely of single males incubating eggs and few nests have failed [13,23,24,31–33,36]. This occurs around one week after peak egg lay, which is the time recommended in the standard methods for the CCAMLR Ecosystem Monitoring Program [37]. At this stage of the breeding season (which in east Antarctica, occurs around late-November to early December) occupied nests are at or near maximal levels (Fig 1). The issue of availability bias arises when the timing of a breeding abundance survey does not coincide with this time of the breeding phenology. Occupied nest counts will under-estimate the metric if they are made earlier in the breeding season before all nests have been established, or later in the breeding season if nests are abandoned due to breeding failures (hence the availability fraction is <1). Sometimes it is not possible to count occupied nests and only counts of adults are possible (e.g. counts from aerial photographs [28]). In a similar way as for occupied nests, some adults will not be available for counting earlier in the breeding season if they have not yet arrived, or later in the season if their breeding attempt has failed and they have left the breeding site. Counts of adults have the additional complication of including both members of a breeding pair and non-breeders at some times of the breeding season. We treat this as an extension of the availability bias issue by assuming that the availability fraction can be >1. Another complication is that many historical abundance surveys have been undertaken late in the breeding season when only counts of chicks are possible. Inferring abundance from chick counts is difficult because chick production is highly variable from year to year [13,24,31].

Most (84%) of the published estimates in the re-construction analysis are unadjusted counts of nests and chicks or adult counts adjusted by a fixed factor (divided by two) (S1 File) and hence do not specifically account for date-specific availability bias. However, there is widespread recognition that the estimates are potentially subject to bias and as such were ‘approximations’ of the breeding population, particularly those estimates based on chick and adult counts [13,24,31].

### Re-constructing abundance estimates

Our approach to re-constructing historical Adélie penguin abundance estimates involved five steps.

#### Step 1. De-constructing historical abundance estimates

We de-constructed published abundance estimates to the critical original raw data (raw count value, precision of the count, date of the count, and what was counted (the latter hereafter referred to as the count object)) by referring to the original publications or reports and searching archived or personal field notebooks.

#### Step 2. Quantifying spatio-temporal variation in adult attendance, nest occupation and chick production/survival

The remote time-lapse camera system developed for Antarctic conditions [38] was used to record the presence of Adélie penguin adults, occupied nests and chicks at sample locations in breeding areas across east Antarctica through multiple breeding seasons. The cameras were deployed under AAS projects 2722, 4086 and 4088 in accordance with permits issued under the Antarctic Treaty (Environmental Protection) Act and approved by the Australian Antarctic Animal Ethics Committee. Photographs obtained from the cameras provided a basis for estimating the extent of contemporary spatial and temporal variation in the presence of adults, occupied nests and chicks at breeding sites, and for developing object-specific and date-specific factors for re-constructing population estimates from the historical count data. Twenty one cameras were deployed at 13 Adélie penguin breeding sites in four regions of the east Antarctica coastline spanning 4,000 km (S2 File). The cameras were deployed between 2007/08 and 2011/12 and collected images continuously from the time of deployment to the last breeding season used in this study (2013/14, although data collection is ongoing). Each camera was positioned to overlook 30–50 breeding nests and programmed to take photographs at solar midday each day across the breeding season from early October to late February.

#### Step 3. Developing a count distribution

We derived a normal distribution of plausible true abundances on the date of the count using the original count value as a mean and a specific interpretation of broad ‘accuracy’ categories provided with published population estimates as a variance. The accuracy categories for published estimates in [13,31] are 1: ±0–5%, 2: ±5–10%, 3: ±10–15%, 4: ±25–50%, 5: order of magnitude. We used the middle of the range for categories 1–4 as a 95% confidence interval. The original descriptions of count objects vary widely in detail and terminology, so we matched each description to one of the three objects counted from camera images (adults, occupied nests or chicks, S1 File).

#### Step 4. Deriving adjustment factors

The methods presented in [26–28] were used to estimate adjustment factors from the time series of images obtained from the cameras. This involved: (1) counting the number of adults, occupied nests and chicks in a fixed region of the photographs, (2) standardising each camera’s seasonal time series count against the maximum number of occupied nests for that camera/breeding season, (3) modelling the standardised seasonal time series counts using a generalised additive model, and (4) given the date, object and location of a count, drawing samples from a pool of modelled seasonal time series to generate date-specific and object-specific factors to adjust population counts. If only a date range was known for the count (e.g. December), samples were drawn across the date range with equal probability. Chick camera counts and adjustment factors were derived only from mid to late January (Fig 1). At this time chicks remain at or close to their nest site and are generally large enough to be highly detectable in camera images (validation work has shown that chick camera counts from single images are unbiased over later January and negatively biased up to mid January [39]). The pool of modelled seasonal time series from which samples were drawn reflected the spatial and temporal variability and uncertainty in applying adjustment factors derived from a small number of breeding sites and breeding seasons to counts over many sites and past seasons. If a historical count was in a region where camera data have been collected, only camera data from that region were included in the pool. If a count was outside the regions with camera data, camera data from all regions were included. Regardless of the count location, the pool included camera data from all years to account for annual variation in adult, nest and chick attendance.

#### Step 5. Re-constructing abundance estimates

In the final step we calculated 1,000 estimates of the maximum number of occupied nests by randomly drawing values from the count and adjustment factor distributions and iteratively calculating the product of the counts and the inverse of the adjustment factors. The distribution of re-constructed estimates was summarised by the median and 95% confidence interval, where a 100.(1-α)% confidence interval was taken as the α/2 and 1-α/2 percentile points. Further details of this procedure are in [26–28].

We decided not to re-construct abundance estimates if some aspects of the de-constructed data were poorly defined or there were insufficient data. These circumstances included 37 cases when the original count value was unbounded (e.g. >10,000), the accuracy was category 5 (order of magnitude), the count object description could not be closely matched to the camera objects, or the count was made outside the date range when adjustment data are provided by cameras.

## Results

### Count data

The re-construction analysis assessed 222 cases of count data from 81 breeding sites across east Antarctica [40]. We viewed original count data (count value, precision, date and object) for 70% of these cases and inferred the original data from descriptions of the estimation method in the remaining 30% of cases. Original descriptions of count objects were variable and potentially subject to interpretation uncertainty when classifying them as adults, occupied nests or chicks (S1 File). The majority of cases (63%) were assigned an adult category, with fewer counts categorised as occupied nests (25%) or chicks (12%). The dates of adult counts extended throughout the breeding season from early October to late February, occupied nest counts were made from early November to late February, and chick counts from mid January to late February (S1 File). We found original data had been erroneously translated or interpreted in 12% of cases when deriving or citing published estimates (S1 File). In these cases we used the original data for re-construction.

### Adjustment data

The cameras provided a pool of 101 complete breeding season time series counts for developing adjustment factors. Standardised counts show a consistent and strong bimodal pattern in the number of adults, a unimodel or weakly bimodal pattern of occupied nests, and no obvious pattern for chicks (Fig 1). The envelopes of standardised count data in Fig 1 characterise the spatio-temporal variation in adult attendance, nest occupation and chick survival/production.

### Re-construction case studies

Three case studies are described below to illustrate the re-construction process for counts of occupied nests, adults and chicks.

#### Case 1. Occupied nest count at Cameron Island, Wilkes Land

The first reported count of Adélie penguins breeding on Cameron Island was ‘approximately 350 occupied nests in 1961’ [41]. The date of the count was not given. The accuracy of the count was subsequently rated as ±10–15% and the population estimated as 350 breeding pairs [31], with the qualification that ‘the most accurate count of breeding pairs is that derived from a count of nests’ and that ‘nest counts are only under-estimates of breeding pairs by the number of breeding failures between egg lay and the date of the count’.

**Fig 2:**
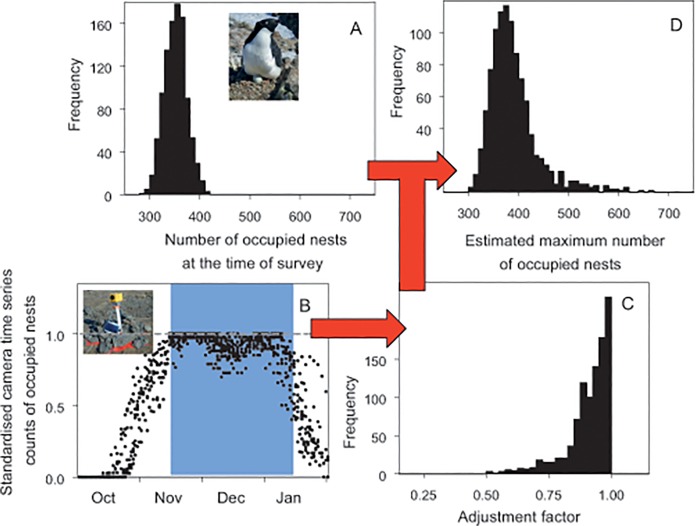
Re-construction process and outcome for a historical count of occupied nests at Cameron Island, east Antarctica. A: distribution of original count data; B: time series counts from time-lapse cameras, with date range of the original count shaded in blue; C: distribution of adjustment factors for the date range of the count, standardised to the maximum number of occupied nests; and D: distribution of estimates of the maximum number of occupied nests.

To re-construct an estimate of the Cameron Island breeding population from this information, we first used the count value (350) and the middle of the count accuracy range (12.5%) as a mean and 95% confidence interval to construct a normal distribution of the number of occupied nests at the time of the count (350, 95% CI 310–396; panel A in Fig 2). As there was no specific information on the date of the count in the original record, we conservatively assumed the count was made between 15 November when nest numbers reach a peak and 15 January when the number of occupied nests starts to decline rapidly as chicks creche (panel B in Fig 2, blue shading). We used standardised time series counts of occupied nests from a pool of 13 camera-location x breeding seasons in the Windmill Islands (panel B in Fig 2) and derived a distribution of adjustment factors relevant to the date range of the count by drawing adjustment data for all dates between 15 November and 15 January (median factor 0.93, 95% CI 0.65–1.00); panel C in Fig 2). Combining the count and adjustment data gave a re-constructed population estimate of 380 (324–533) occupied nests (panel D in Fig 2).

#### Case 2. Adult count at Rookery Island, Mac.Robertson Land

**Fig 3:**
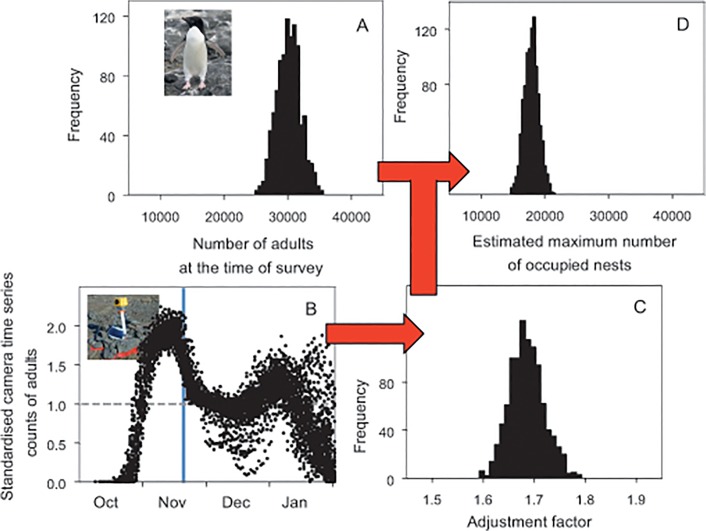
Re-construction process and outcome for a historical count of adults at Rookery Island, east Antarctica. A: distribution of original count data; B: time series counts from time-lapse cameras, with date of original count shaded in blue; C: distribution of adjustment factors for the date of the count, standardised to the maximum number of occupied nests; and D: distribution of estimates of the maximum number of occupied nests.

The Adélie penguin population breeding at Rookery Island in the 1972/73 breeding season is estimated to have been 15,020 breeding pairs [31]. The estimate is derived from an unspecified count of adults on 17/11/72 with an accuracy rated as ±10–15%. We were unable to view the original count record, and assumed an original count of 30,040 adults based on the statement that ‘counts of birds or adults which appear unqualified in log books have been divided by two to give an estimate of the number of breeding pairs’ [31]. As in case 1, we re-constructed the count data by taking the raw count of 30,040 as the most likely number of adults present at the time of the count and using the middle of the count accuracy range (±12.5%) as a 95% confidence interval (panel A in Fig 3). Using standardised time series count data of adults for the date of the count from a pool of 60 camera-location x breeding seasons on the Mac.Robertson Land coast, we estimated the adjustment factor as 1.68 (1.62–1.76) (panel C in Fig 3). A re-constructed population estimate obtained by combining the count and adjustment data was 17,918 (15,563–20,234) occupied nests (panel D in Fig 3).

#### Case 3. Chick count at Mackellar Islands, George V Land

There are three historical records or observations of the Adélie penguin breeding population at the remote Mackellar Islands in George V Land. The 1911–14 and 1929–31 BANZARE expedition reports [42] describe two apparently brief visits to these islands on 18 December 1913 and 6 January 1931, noting on the first visit that ‘…from Hunter’s log…we estimated there must be 200,000 penguins on the islands…’ and subsequently that ‘…Hunter’s estimate of 200,000 birds is conservative, and there might prove to be twice that number if a full survey were undertaken’. Although these observations were subsequently used to estimate populations of 100,000 and 200,000 breeding pairs respectively [31], we did not attempt to re-construct population estimates because the count observations appear to lack rigour.

The third visit to the Mackellar Islands was on 13–14 January 1981 and a rigorous survey effort returned a count of 27,260 chicks [43]. The count was subsequently rated as accurate to ±0–5% [13] and a minimum estimate of 27,260 breeding pairs was proposed [13] on the notion that most pygoscelid penguins raise one chick per pair per season [24,31]. The estimation method was qualified by noting that interpreting chick counts is subject to uncertainties related to temporal variation in breeding effort and success and would be a minimum estimate [13].

**Fig 4:**
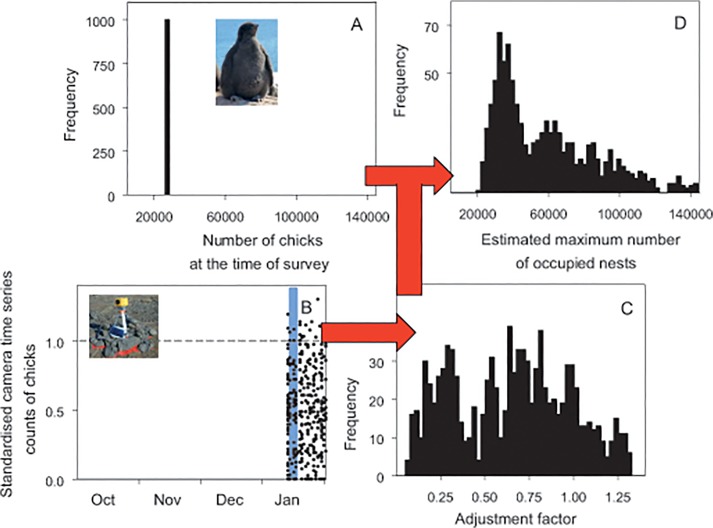
Re-construction process and outcome for a historical count of chicks at Mackellar Islands, east Antarctica. A: distribution of original count data; B: time series counts from time-lapse cameras, with date range of original count shaded in blue; C: distribution of adjustment factors for the date range of the count, standardised to the maximum number of occupied nests; and D: distribution of estimates of the maximum number of occupied nests. Although chicks are present at the breeding site from late December when they hatch to late February when they leave the site to begin foraging, time series counts in panel B were only made from mid to late January when chicks remain at or close to their nest site and are large enough to be highly detectable in camera images.

Using the information in [13,31], a re-constructed count was 27,260 (26,580–27,987) chicks (panel A in Fig 4). Although we have deployed a camera at nearby Cape Denison (S2 File), we have not yet been able to download images and instead used data obtained from cameras deployed in all other regions across east Antarctica to derive a distribution of chick adjustment factors specific to the dates of the count (0.57 (0.12–1.27), panel C in Fig 4), after adjusting for slight under-counting of chicks from images taken in mid January). Combining the count and adjustment data gave a re-constructed population estimate of 54,472 (25,110–170,153) occupied nests (panel D in Fig 4).

### Reconstruction analysis

**Fig 5:**
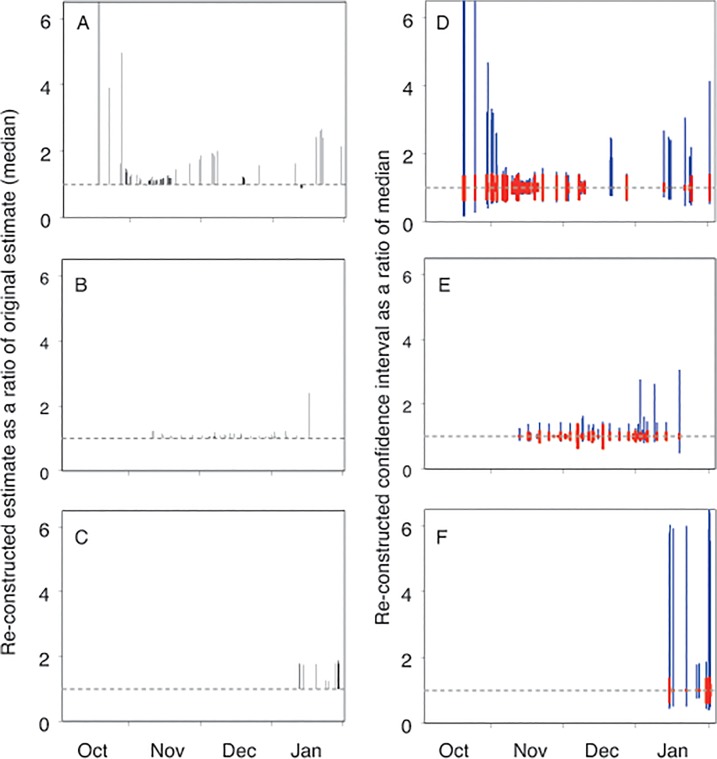
Comparison of re-constructed and published historical abundance estimates relative to the date of the count. Left panels: Re-constructed median estimates expressed as a ratio of the published estimates for adults (A), nests (B) and chicks (C). Values >1 indicate re-constructed estimates are larger than published estimates (i.e. published estimates are under-estimates). Right panels: Re-constructed 95% confidence intervals expressed as a ratio of the re-constructed median values for adults (D), nests (E) and chicks (F). Red bars are the contribution of uncertainty in the count, and blue bars are the additional contribution to uncertainty associated with adjusting the raw count to a standard population metric. The dashed grey horizontal lines at unity indicate (A-C) no difference between re-constructed and published estimates and (D-F) no uncertainty around the re-constructed estimates.

We re-constructed 185 of the 222 published abundance estimates. There was a strong tendency (90% of cases) for re-constructed estimates to be higher than original published estimates (panels A-C in Fig 5). This pattern was most pronounced when counts were made early and late in the breeding season, when some re-constructed estimates were up to 10 times higher than original published estimates. Estimates that were re-constructed from counts made in November and December were on average 1.19 times higher (range 1.00–2.01) than their equivalent published estimates. Regardless of the timing of counts, the tendency for re-constructed counts to be higher than original estimates was greatest for adult counts, smallest for nest counts, and intermediate for chick counts.

Confidence intervals around re-constructed estimates were also higher for counts of adults made early and late in the breeding season and counts of chicks late in the breeding season (panels D-F in Fig 5). At these times the dominant contribution to the overall confidence interval was related to spatio-temporal variation in adult attendance or chick production/survival and its effect on uncertainty in the adjustment process. In contrast, the confidence intervals for estimates re-constructed from counts of adults and nests made in November and December were smaller and dominated by uncertainty in the count.

## Discussion

Our study highlights the importance of accounting for biases and uncertainties when estimating animal abundance from historical counts. By collecting new adjustment data with remotely-operating cameras and incorporating these data in the estimation process, we have demonstrated that many published historical abundance estimates for east Antarctic Adélie penguins are under-estimates (negatively biased) and have unrealistically low levels of uncertainty given the count and adjustment data from which they were derived. These results are consistent with qualifications that researchers have given to historical estimates. For example, the frequent practise of using unadjusted chick counts as an approximate estimate of breeding pairs when counts are only possible late in the breeding season has usually been accompanied by a statement noting that such estimates are difficult to interpret because of wide annual fluctuations in breeding success [13,24,31]. Similarly, it has often been noted that unadjusted counts of nests will under-estimate breeding abundance if some breeding attempts fail before a count occurs, but nevertheless counts of nests are often recommended as providing a more reliable basis for estimating breeding abundance than counts of adults or chicks. Our work captured this widespread qualitative recognition and expressed it in quantitative terms.

These results have important implications for conclusions about long-term population change that use original historical estimates as a base for inference. Given that our re-constructions showed that most of the original published estimates are negatively biased, their use to assess population change could exaggerate the rate of a real increase or mask the detection of a real decrease. Consistent with this expectation, some of the conclusions reached in a recent global assessment of Adélie penguin population change based on published historical estimates and recent satellite-based estimates [29] are altered when the published historical estimates are replaced with our re-constructed estimates. In the seven cases from the global assessment that were included in our re-constructions, three conclusions of ‘no detectable change’ are the same (Chick Island, Lewis Island and Rookery Islands), but three conclusions of ‘increase’ are downgraded to ‘no detectable change’ or ‘likely increase’ (Cape Denison, Mackellar Islands and Way Archipelago). The three downgraded cases pertain to estimates based on counts of chicks along the George V Land coast [43]. Our re-constructions returned estimates with median values higher than published estimates and with substantially wider confidence intervals. These differences can be attributed to the level of breeding success across east Antarctica revealed by the cameras being lower than the one-chick-per-nest assumption in the original estimation process, and to the annual variation in chick production being propagated through to a high uncertainty in the abundance estimate. In contrast, although our re-constructed historical estimates for six breeding sites near Mawson station (the 7^th^ case from the global assessment: Mawson coast) were 1.14–1.22 times higher than published estimates, our assessment upgrades the conclusion of change for this region from ‘no detectable change’ to ‘increase’. In this case the change is due to confusion over which breeding sites were included in the historical estimate for the Mawson coast (S1 File).

**Fig 6:**
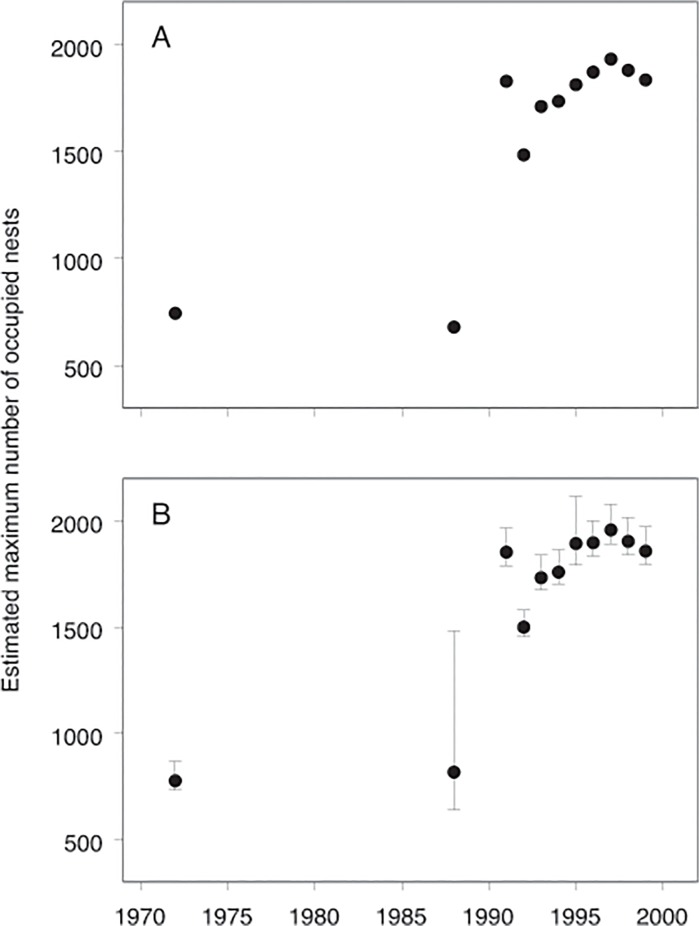
Comparison of (A) published and (B) re-constructed time series abundance estimates for Béchervaise Island, east Antarctica. Solid circles indicate median values, and error bars are 95% confidence intervals. The published time series is from [30].

Our results also have implications for interpreting the form of long term population change. Although the conclusions of a long term increase in Adélie populations breeding at two sites in east Antarctica (Béchervaise Island and Whitney Point) in [30] are essentially unchanged when replaced with our re-constructed estimates, the re-constructed estimates suggest that an alternate interpretation of the form of change at Béchervaise Island is plausible. The original data suggest a step-wise doubling of the population from 1988/89 to 1991/92 (panel A in Fig 6) which was interpreted as indicating ‘non-linear population dynamics’ [30]. Our re-construction of the 1988/89 count produced an estimate with a large, upwardly skewed uncertainty, while estimates for all other years have relatively small, symmetrical uncertainties (panel B in Fig 6). A consequence of the re-construction is that both step-wise and steady increases are plausible interpretations of the time series. Importantly, these different interpretations can lead different conclusions about the underlying population dynamics and the likely ecological causes. The basis of this outcome relates to the timing of the counts (late December in 1988/89, early December in all other breeding seasons) and new observations from the cameras of high nest attrition through December in some years in the region where Béchervaise Island is located (Mac.Robertson Land, left middle panel in Fig 1). Thus it is possible that the 1988/89 population count in late December occurred after high nest attrition and departure of breeders from the site, resulting in the count being lower than it would have been if made in early December when the other time series counts were made.

Our search for original data records revealed some errors in translation through the publication process and a general drift towards apparent higher certainty in abundance estimates as the estimates were successively cited. Both of these processes could affect conclusions about population change. The original published description of counts at Mt Biscoe in east Antarctica, for example, states that on a short visit on 27 October 1985 ‘only six Adélie penguins were seen’, on a subsequent visit of 1.5 hours on 29 October ‘hundreds of birds were present’, and lastly that ‘the colony was estimated to contain *at least* 5,000 breeding pairs’ from the extent of guano measured from photographs [44]. An estimate of 5,000 breeding pairs for this site was subsequently included in an Antarctic-wide compendium of minimum estimates [13], an unqualified estimate of 5,000 breeding pairs was then included in a revised Antarctic-wide compendium [45], and finally the unqualified estimate of 5,000 breeding pairs was compared against a recent satellite-based estimate to conclude that the population was ‘increasing’ [29]. We declined to re-construct an abundance estimate for Mt Biscoe from the original count values because they were made very close to or on the day of first arrival or were only roughly described. We also note that re-constructed estimates based on counts made this early in the breeding season have high uncertainty related to annual variation in the time of arrival at the breeding site that will make detection of change difficult (panel D in Fig 5). We encourage any future analyses of penguin population change to wherever possible use original count data, and to this end we collated a summary of original descriptions of counts used for our re-construction analysis in [40].

Estimating availability bias is one of the more difficult aspects of abundance surveys for any species because it requires collection of additional data using methods external to the counting effort [9]. Although remote photography has a long history in ecological research [46] and has been used to study a wide range of issues in behavioural ecology including the foraging ecology of penguins [47], there has been relatively little use of cameras to estimate population parameters or understand population dynamics. This situation is now changing as ecologists become increasingly aware of the potential use of cameras for advancing animal population studies [48,49]. For penguins, remotely operating time-lapse cameras have facilitated a quantum leap in the collection of breeding phenology and attendance data for availability adjustments to population counts. This improved data collection ability will ensure that adjustment models are not constrained by the amount and resolution of data. This was an issue in the development of hierarchical Bayesian models for adjusting nest counts in the Antarctic Peninsula, when the coarse temporal resolution of available nest attrition data ruled out consideration of non-linear models [25]. Cameras also make simultaneous observations of adults, nest and chicks, allowing adjustment models to be developed for counts of any of these population objects.

The seasonal time series data obtained from cameras presented here indicate there is substantial spatial and temporal variation in Adélie penguin presence and attendance at breeding sites, and therefore in availability bias. Heterogeneous availability may be a widespread phenomenon in animal abundance surveys, but few studies have been able to quantify or assess heterogeneity because of the difficulty in collecting availability data [50]. This heterogeneity has consequences for interpreting historical abundance data, because without coincident collection of availability data at the time and place of historical counts, it is not possible to know in retrospect which specific adjustment factors to apply from the measured spatio-temporal range. In the absence of this knowledge, we took a precautionary approach by drawing availability data from a pool of attendance curves representing spatio-temporal variation and propagated this uncertainty through to the re-constructed abundance estimates.

With the cameras now established and operating remotely, the body of availability data will increase rapidly with minimal effort, allowing better insights on the influence of environmental conditions such as sea-ice extent and weather on the attendance and breeding success of Adélie penguins at their breeding sites. In the near future it may be possible to develop predictive models relating variation in availability data to environmental covariates (e.g. [16,50]), and use these models to reduce the uncertainty in some re-constructed historical abundance estimates. Alternatively, it may be possible to truncate the upper limits of large uncertainties by taking into account information additional to the count and camera data such as the area of suitable breeding habitat at each site. However, our work demonstrates that significant reductions in uncertainty are only likely to be possible for counts made early or late in the breeding season when overall uncertainty is largely dominated by the adjustment process. Uncertainty in historical estimates derived from counts in the middle of the breeding period is dominated by the precision of the existing counts which cannot be improved.

In addition to providing a large body of new data on daily changes in colony attendance, the time-lapse cameras also have the potential to provide data on diel changes in attendance. This can be done by re-programming the cameras to take multiple photographs each day. Any consistent and significant within-day variation in colony attendance could affect abundance estimation. While it would not be possible to adjust historical counts for time of day effects because no time data were collected in the past, the uncertainty could be accounted for by incorporating this variation into the adjustment curves.

In colonial-breeding species, availability bias is time-dependent and strongly related to breeding phenology. In this situation, using contemporary availability data to adjust counts obtained decades ago could result in biased abundance estimates if phenology has changed substantially over the intervening period. In the northern hemisphere, analyses of long-term phenology data have revealed significant advances in the timing of spring breeding events such as arrival of many migrant bird species [51]. In east Antarctica, changes in Adélie penguin breeding phenology appear to be limited and unlikely to lead to significant biases when re-interpreting historical count data: the dates of Adélie penguin arrival and egg lay in Adélie Land have been delayed at rates of 0.4 and 0.8 days decade^-1^ respectively over the past 50 years [52], and there has been no significant change in Adélie penguin breeding phenology over a 16-year period from the early 1990s in Mac.Robertson Land [53]. Nevertheless, historical phenology data are sparse and we recommend that efforts are made to search for data that are consistent in nature and form with the camera data to more fully investigate the extent and magnitude of possible past changes in phenology, so that any changes can be incorporated into adjustment of historical count data if necessary. Without such data, the effect of any past change in phenology on abundance estimates will be unknown. The development of time-lapse cameras will allow any future changes in phenology to be quantified and taken into account in future population monitoring.

The approach taken in this study for Adélie penguins could be applied more broadly to a wide range of colonial, land-breeding marine species including other penguins, flying seabirds and marine mammals. The common life history characteristics of these species’ groups that makes these methods appropriate and amenable are (1) their tendency to form large, dense breeding aggregations, making large numbers of animals visible to observations from a fixed point, (2) their variable attendance at colonies within the breeding season, leading to the potential for population counts to be biased because of varying availability of animals to detection in population surveys at colonies, and (3) the remoteness of many breeding colonies and the difficulty that researchers may have in accessing the colonies for population counts at an optimal time. Re-assessing historical data for these species’ groups using the methods described here would help to ensure that reliable inferences are made about the nature, form and cause of past changes in marine populations and ecosystems.

## Supporting Information

S1 File Details on historical population counts and estimates. (DOC)

S2 File Locations of remotely operating time-lapse cameras. (DOC)
