# Supplementary material for: Re-Constructing Historical Adélie Penguin Abundance Estimates by Retrospectively Accounting for Detection Bias
Source: PLoS One. 2015 Apr 24;10(4):e0123540. doi: 10.1371/journal.pone.0123540 (PMC4409151; doi:10.1371/journal.pone.0123540)
Supplement: S1 File — (DOC) [file pone.0123540.s001.doc]

**Figure A. Timing of historical counts of adults, occupied nests and chicks**.


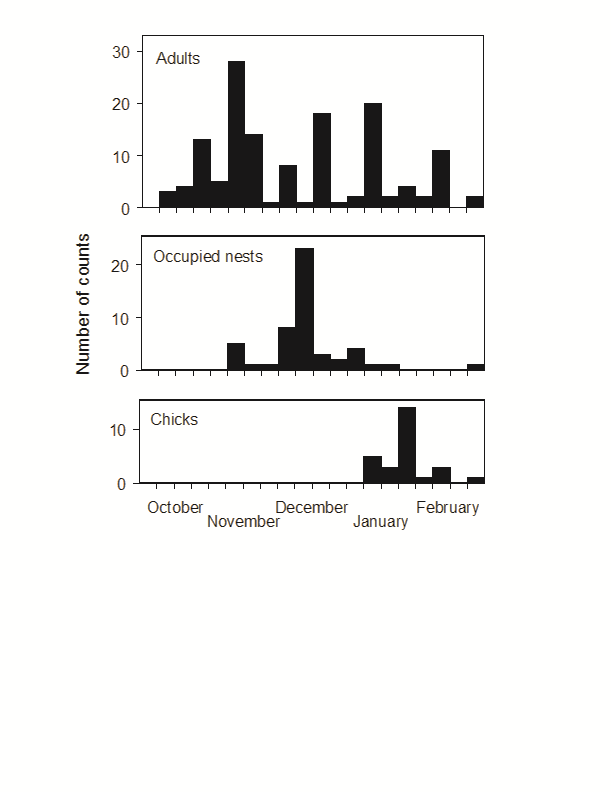


| **Table A. Methods used to derive original population estimates**   | **Source** | **Number of cases**  **that were re-constructed** | **Estimation method** | | --- | --- | --- | | Horne (1983) | 136 | Counts of nests and chicks were taken without adjustment as the estimated number of breeding pairs. Counts of birds or adults that were unqualified in log books were divided by two to estimate the number of breeding pairs | | Martin et al. (1990) | 1 | A count of the number of incubating birds in mid December was taken without adjustment as an estimate of the number of breeding pairs | | Alonso et al. (1987) | 2 | A count of chicks in early February was adjusted with an average productivity value (chicks/nest) from another region and year to estimate the number of breeding pairs | | Bassett et al (1990) | 1 | A conservative estimate of the breeding population was derived by mapping the area of guano present in late October and assuming a minimum nesting density within the colony | | Cooper (1985) | 1 | An estimate of the breeding population was derived by roughly pacing around the perimeter of the colony and assuming a nesting density within the colony | | Ensor and Bassett (1987) | 15 | Counts of chicks made in mid-late January were presented unadjusted in Ensor and Bassett and later interpreted in Woehler (1993) as a minimum estimate of the number of breeding pairs | | Woehler (1989a) | 23 | Counts of all adults made in early-mid November were divided by two to estimate the number of breeding pairs | | Woehler (1989b) | 34 | Counts of adults in December and January were adjusted using weekly counts of adults and nests in a sample of sub-colonies in another region and year to estimate the number of breeding pairs | | | |
| --- | --- | --- | --- | --- | --- | --- | --- | --- | --- | --- | --- | --- | --- | --- | --- | --- | --- | --- | --- | --- | --- | --- | --- | --- | --- | --- | --- | --- | --- |
|  | | |
|  | | |
|  |  |  |

**Table B. Description of count objects in original reports and matching objects for camera counts**.

| **Original description** |  |
| --- | --- |
| Adults | Adult |
| Birds | Adult |
| Breeding birds | Adult |
| Heads | Adult |
| Individuals | Adult |
| Breeding pairs | Occupied nests |
| Incubating birds | Occupied nests |
| Incubating and brooding birds | Occupied nests |
| Incubating nests | Occupied nests |
| Nests | Occupied nests |
| Nesting pairs | Occupied nests |
| Nesting sites | Occupied nests |
| Occupied nests | Occupied nests |
| Pairs | Occupied nests |
| Mating pairs | Occupied nests |
| Chicks | Chicks |

**Table C. Inconsistent or erroneous translation of count data from original descriptions to original or cited published estimates.** Differences are bolded. Only original descriptions were used for re-constructions.

| **Location and breeding season** | **Original description** | **Published description** |
| --- | --- | --- |
| Forbes Glacier 1971/72 | Count of 213 chicks on **14/2/72** (Kerry field notes) | Count of 213 chicks on **11/2/72** (Horne 1983) **(date incorrect)** |
| Bechervaise Island 1971/72 | Count of 550 **incubating and brooding birds** (Kerry field notes) | Estimate of 550 breeding pairs from a count of **adults** reported (Horne 1983) **(count object differs)** |
| Bechervaise Island 1971/72 | Count of 744 **incubating and brooding birds** (Kerry field notes) | Estimate of 744 breeding pairs from a count of **adults** reported (Horne 1983) **(count object differs)** |
| Verner Island 1971/72 | Count of 1850 **incubating and brooding birds** on 3/1/72 (Kerry field notes) | Estimate of 1850 breeding pairs from a count of **adults** reported on 3/1/72 (Horne 1983) **(count object differs)** |
| Verner Island 1972/73 | Count of 1966 **incubating and brooding birds** on 8/12/72 (Kerry field notes) | Estimate of 1966 breeding pairs from a count of **adults** reported on 8/12/72 (Horne 1983) **(count object differs)** |
| Petersen Island 1971/72 | Count of 643 **incubating and brooding birds** on 3/1/72 (Kerry field notes) | Estimate of 643 breeding pairs from a count of **adults** reported on 3/1/72 (Horne 1983) **(count object differs)** |
| Petersen Island 1972/73 | Count of 500 **incubating and brooding birds** on 8/12/72 (Kerry field notes) | Estimate of 500 breeding pairs from a count of **adults** reported on 8/12/72 (Horne 1983) **(count object differs)** |
| Welch Island 1971/72 | Count of 3000 **incubating and brooding birds** on 2/1/72 (Kerry field notes) | Estimate of 3000 breeding pairs from a count of **adults** reported on 2/1/72 (Horne 1983) **(count object differs)** |
| Welch Island 1972/73 | Count of 9445 **incubating and brooding birds** on 8/12/72 (Kerry field notes) | Estimate of 9445 breeding pairs from a count of **adults** reported on 8/12/72 (Horne 1983) **(count object differs)** |
| Klung Island 1972/73 | Count of 4419 **incubating and brooding birds** on 8/12/72 (Kerry field notes) | Estimate of 4419 breeding pairs from a count of **adults** reported on 8/12/72 (Horne 1983) **(count object differs)** |
| Giganteus Island 1971/72 | Count of 4850 **incubating and brooding birds** (Kerry field notes) | Count of 4850 **chicks** reported (Horne 1983) **(count object differs)** |
| Tangholmane Island 1973/74 | Count of **>1900** **birds** **(parents plus chicks)** on 2/3/74 (Davis biology log) | Count of **2000** **adults** on 2/3/74 (Horne 1983) **(‘greater than’ qualification lost, count object differs)** |
| Mule Island 1976/77 | Count of **5000-6000** birds on 4**/**11/76 (Davis biology log) | Count of **3000** adults reported on 4**/**11/76 (Horne 1983) **(count value differs)** |
| Island west of Redfearn Island 1963/64 | Count of 12000-13000 birds on **7/12/63** (Davis biology log) | Estimate of 6500 BP (A3) from a count on **17/12/63** (Horne 1983) **(date incorrect)** |
| Warriner Island 1970/71 | Count of 20000 Adelies on **6/12/70** (Davis biology log) | Estimate of 10000 BP (A4) (Horne 1983) from a count on **15/11/70 (date incorrect)** |
| Chappel Island 1972/73 | Count of approximately **14000** (unspecified objects) on 7/11/72 (Casey biology log) | Estimate of **8000** BP (A3) in 1972 (Horne 1983) **(count value differs)** |
| Shirley Island 1970/71 | Count of 7580 nest sites on **7/12/71** (Casey biology log) | Estimate of 7580 BP (N1) from a count on **7/2/71** (Horne 1983) **(date incorrect)** |
| Shirley Island 1971/72 | Count of 4336 chicks on 27/1/72 (Casey biology log, Horne 1983) | Estimate of 4366 BP (C1) from a count on 27/1/73 (Woehler et al. 1991) (**count and date incorrect**) |
| Shirley Island 1972/73 | Count of 9687 chicks on **26/1/73** (Casey biology log) | Estimate of 9687 BP (C1) from a count on **26/1/72** (Horne 1983) **(date incorrect)** |
| Shirley Island 1976/77 | Count of 7362 **nests sites** on 26-28/11/76 (Casey biology log) | Estimate of 7362 BP (A1) from a count of **adults** on 26-28/11/76 (Horne 1983) **(count object differs)** |
| Whitney Point 1961/62 | Count of **1300** occupied nests in 1961 (Orton 1963) | Estimate of **4800** BP in 1961 (N2) (Horne 1983). **The value of 4800 is the sum of Whitney and Blakeney Points (1300 + 3500) from Orton (1963)** |
| Blakeney Point 1961/62 | Count of **3500** occupied nests in 1961 (Orton 1963) | Estimate of **4800** BP in 1961 (N2) (Horne 1983). **The value of 4800 is the sum of Whitney and Blakeney Points (1300 + 3500) from Orton (1963)** |
| Chick Island 1961/62 | Count of **240** **adults including yearlings** (Casey biology log) | Estimate of **120** BP (A1) from a count of **adults** on 21/1/62 (Horne 1983) **(count object differs)** |
| Magnetic Island 1970/71 | Count of **>10000** Adelies on 6/12/70 (Davis biology log) | Estimate of 5000 BP (A4) on 6/12/70 (Horne 1983)  **(‘greater than’ qualification lost)** |
| Mt Biscoe 1985/86 | Estimate of **>5000** breeding pairs (Bassett et al. 1987) | Estimate of **5000** breeding pairs (Ainley 2002, Schwaller et al. 2013, Lynch and LaRue 2014) **(‘greater than’ qualification lost)** |
| Svenner Islands and Islands north east of Brattstrand Bluffs 1981/82 | Estimate of **23324** breeding pairs (Whitehead and Johnstone 1990) | Estimate of **41389** breeding pairs (Ainley 2002, Schwaller et al. 2013, Lynch and LaRue 2014) (**41389 estimate includes estimate of 18065 for Svenner Islands twice**) |
| Mawson ‘coast’ or ‘area’ 1988/89 and Gibbney Island 1981/82 | Estimate of 16950 breeding pairs for six islands combined close to Mawson station in 1988-89 (Bechervaise, Verner, Petersen, Welch, Klung and island west of Klung) and 20745 for Gibbney Island and Island near Forbes Glacier combined in 1981-82 (Woehler et al. 1989). These estimates appear in a section of Table 1 of Woehler et al. (1989) titled ‘Mawson area’ which includes all the above sites but no others. | Collectively all 8 islands are referred to as the ‘Mawson area’ and the total population estimated as 37695 breeding pairs in Woehler (1993). The supplementary table in Lynch and LaRue (2014) has separate entries for Mawson coast, Gibbney Island and Forbes Glacier. The entries for ‘previous estimates’ in this table for the Mawson coast and Gibbney island are ‘**37695**’ and ‘**no previous estimate**’ (**the Mawson coast estimate of 37695 incorrectly includes Gibbney and Forbes Glacier and should be 16950; the entry for previous estimate for Gibbney is ‘no previous estimate’ and should be 19324**). |
