# Supplementary material for: Re-Constructing Historical Adélie Penguin Abundance Estimates by Retrospectively Accounting for Detection Bias
Source: PLoS One. 2015 Apr 24;10(4):e0123540. doi: 10.1371/journal.pone.0123540 (PMC4409151; doi:10.1371/journal.pone.0123540)
Supplement: S2 File — (DOC) [file pone.0123540.s002.doc]

**Figure A. Location of 21 remotely operating time-lapse cameras across east Antarctica**

**
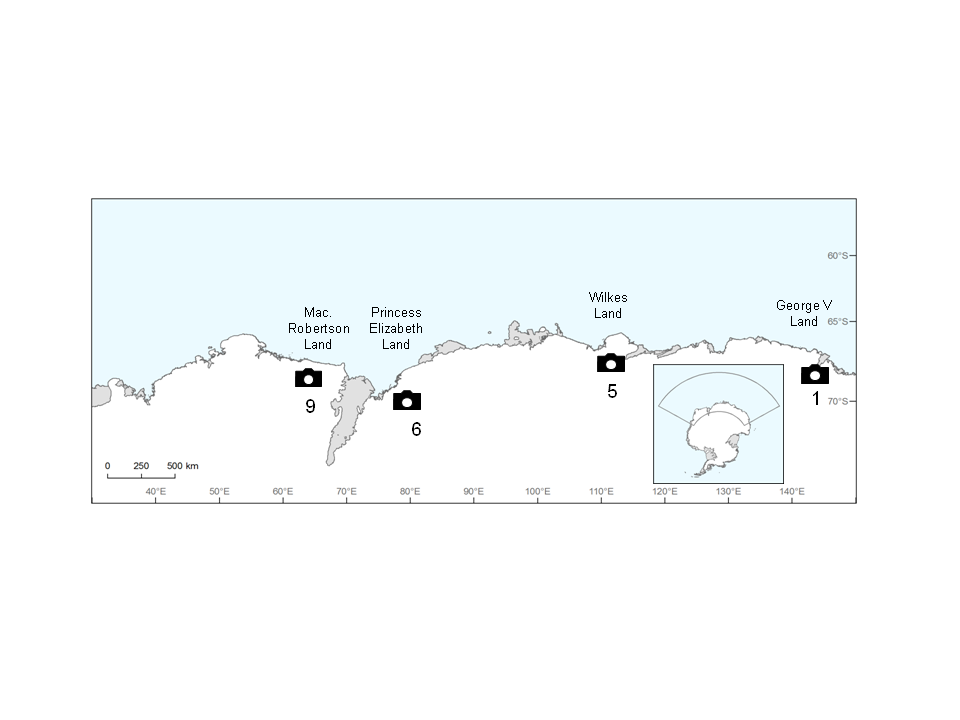
**

|  | | |
| --- | --- | --- |
|  | | |
|  | | |
|  |  |  |

**Table A. Location of 21 remotely operating time-lapse cameras across east Antarctica**. A breeding site is a discrete island or outcrop of continental rock where Adélie penguins breed. Multiple cameras at the same site are located at different sub-colonies.

| **Region** | **Breeding site** | **Latitude** | **Longitude** |
| --- | --- | --- | --- |
| Mac.Robertson Land | Béchervaise Island | -67.58646 | 62.81107 |
| Mac.Robertson Land | Béchervaise Island | -67.58756 | 62.81156 |
| Mac.Robertson Land | Welch Island | -67.55598 | 62.91587 |
| Mac.Robertson Land | Welch Island | -67.55588 | 62.91805 |
| Mac.Robertson Land | Welch Island | -67.55706 | 62.91588 |
| Mac.Robertson Land | Petersen Island | -67.57834 | 62.88763 |
| Mac.Robertson Land | Petersen Island | -67.57826 | 62.88743 |
| Mac.Robertson Land | Verner Island | -67.57609 | 62.88341 |
| Mac.Robertson Land | Un-named island | -67.63210 | 62.51331 |
| Princess Elizabeth Land | Gardner Island | -68.57922 | 77.86666 |
| Princess Elizabeth Land | Gardner Island | -68.57946 | 77.87063 |
| Princess Elizabeth Land | Gardner Island | -68.57888 | 77.86636 |
| Princess Elizabeth Land | Magnetic Island | -68.54358 | 77.90379 |
| Princess Elizabeth Land | Magnetic Island | -68.54346 | 77.90406 |
| Princess Elizabeth Land | Hop Island | -68.82360 | 77.68037 |
| Wilkes Land | Whitney Point | -66.25158 | 110.53058 |
| Wilkes Land | Whitney Point | -66.25089 | 110.53406 |
| Wilkes Land | Blakeney Point | -66.24228 | 110.58144 |
| Wilkes Land | Shirley Island | -66.28250 | 110.48569 |
| Wilkes Land | Odbert Island | -66.37717 | 110.56536 |
| George V Land | Cape Denison | -67.01090 | 142.68117 |
